# Genetic link between family socioeconomic status and children's educational achievement estimated from genome-wide SNPs

**DOI:** 10.1038/mp.2015.2

**Published:** 2015-03-10

**Authors:** E Krapohl, R Plomin

**Affiliations:** 1 King's College London, MRC Social, Genetic and Developmental Psychiatry Centre, Institute of Psychiatry, Psychology & Neuroscience, London, UK

## Abstract

One of the best predictors of children's educational achievement is their family's socioeconomic status (SES), but the degree to which this association is genetically mediated remains unclear. For 3000 UK-representative unrelated children we found that genome-wide single-nucleotide polymorphisms could explain a third of the variance of scores on an age-16 UK national examination of educational achievement and half of the correlation between their scores and family SES. Moreover, genome-wide polygenic scores based on a previously published genome-wide association meta-analysis of total number of years in education accounted for ~3.0% variance in educational achievement and ~2.5% in family SES. This study provides the first molecular evidence for substantial genetic influence on differences in children's educational achievement and its association with family SES.

## Introduction

After health care, education is society's largest and most expensive environmental intervention, consuming >6% of gross domestic product in OECD (Organization for Economic Co-operation and Development) countries.^1^ Understanding the etiology and correlates of differences between children in what they take away from their education is important because their educational achievement directly determines admission to further education and employability and also predicts a wide range of health outcomes.^1, 2, 3^ Pedigree-based methods, primarily twin studies comparing the similarity of identical and nonidentical twins, have consistently suggested substantial genetic influence on differences between children in their educational achievement.^4, 5, 6, 7, 8, 9, 10^ It is now possible to use DNA-based methods to estimate genetic influence on variance in large samples of unrelated individuals.^11, 12^ No DNA-based estimates of genetic influence have as yet been reported for children's educational achievement, although evidence has been reported for the rough proxy of total number of years in education in adults.^13, 14, 15, 16^ This study used children's genotypes to estimate genetic influences on variance in educational achievement and its covariance with family socioeconomic status (SES).

Here we report the first investigation of genetic influence on the variance of children's educational achievement using DNA alone. The same DNA-based methods can also be used to estimate genetic influence on the covariance between traits.^17^ This enabled us to investigate possible genetic mediation of the best predictor of children's educational achievement, their family's SES.^18, 19^ This correlation is often interpreted causally as family SES causing differences in children's educational achievement.^20^ However, it remains unclear whether and to what extent the association between family SES and children's educational achievement is genetically mediated, because twin and family research is limited to studying phenotypes that can vary within a family. Key aspects of children's environment such as poverty, parental education and neighborhood cannot be investigated using the twin method because it is methodologically impossible to decompose variance in phenotypes shared within twin pairs.

The DNA-based technique, genome-wide complex trait analysis (GCTA),^11^ fits the effects of genome-wide single-nucleotide polymorphisms (SNPs) as random effects in a mixed linear model to estimate variance or covariance captured by all SNPs simultaneously. Contrary to traditional family-based methods that estimate the genetic contribution to phenotypic variation or covariation by known kinship coefficients, GCTA relies on empirical genetic resemblance established from identity by state inferred from genome-wide SNP similarity of ‘unrelated' individuals.

Because GCTA is based on unrelated individuals, it enables the decomposition of variance of phenotypes such as family SES that are the same for members of a family and therefore cannot be decomposed in analyses such as the twin method that rely on within-family differences. Another difference between the two methods is that, unlike the twin method, GCTA is limited to estimating additive genetic effects for the SNPs on the genome-wide DNA array or other DNA variants in linkage disequilibrium with the measured SNPs, which until recently have been common SNPs. Thus, GCTA will underestimate genetic influence to the extent that nonadditive effects or rare variants contribute importantly to heritability. This limitation of GCTA to additive effects of common SNPs is the same limitation of genome-wide association (GWA) studies that attempt to identify specific SNPs associated with a trait. GCTA is directly comparable to GWA results because both rely on the same experimental design using the same genetic signal;^21^ GCTA provides an upper-limit estimate of the genetic effects that can be identified by GWA.

GWA attempts aimed at identifying individually significant SNPs have generally captured only extremely small fractions of genetic variance of complex traits, the so-called *missing heritability* problem.^22^ However, evidence has been accumulating that significant portions of phenotypic variation can be explained by the ensemble of markers not achieving genome-wide significance.^23^ Markers are identified from GWAs using an initial discovery sample to construct a genome-wide polygenic score (GPS) in an independent replication sample by calculating the effect-size-weighted sum of trait-associated alleles for each individual. An aggregate GPS score can be used to assess genetic influence on trait variation.

As they are tapping into the same genetic signal, GPS based on GWA results and GCTA can be applied to the same data sets, with both estimating the polygenic contribution to trait variance or a shared polygenic covariance between traits captured by the additive effects of common SNPs. We therefore employ a two-method approach using GCTA and GPS to explore the genetic influence on the variance of children's educational achievement and on the covariance between family SES and children's educational achievement. Our study had four objectives:

(1) To estimate, for the first time using DNA data, genetic influences on children's educational achievement on an age-16 UK national examination of educational achievement using genome-wide genotypes from >3000 conventionally unrelated children. Specifically, we conduct GCTA^11^ to quantify pairwise genomic similarity between each pair of individuals across millions of SNPs throughout the genome in order to estimate the proportion of phenotypic variation in children's educational achievement captured by all SNPs simultaneously.

(2) To investigate genetic mediation of the phenotypic correlation between family SES and children's educational achievement, we conduct bivariate GCTA to estimate the proportion of phenotypic covariation between children's family SES and children's educational achievement captured by children's genotypes.

(3) To create a GPS based on the results of a large GWA study on adults' total years of schooling^13^ and investigate its association with variance in children's educational achievement and their family SES.

(4) To examine the role of general cognitive ability (intelligence) in the genetic nexus between children's educational achievement and their family SES. Molecular evidence as well as twin studies have shown that cognitive ability is heritable and accounts for substantial portion of genetic variance in educational achievement.^7, 24, 25, 26^ In addition, recent molecular evidence from the present sample of unrelated individuals showed high genetic correlation between family SES and children's intelligence at age 7 and 12 years.^27^ Based on this evidence, it is important to address the question to what extent the genetic link between family SES and children's educational achievement is mediated by intelligence. For this reason, we perform GCTA mediation analyses to test for a direct genetic link between family SES and children's educational achievement independent of cognitive ability. Complementarily, we test whether the GPS of adults' total years of schooling explains variance in children's educational achievement independently of cognitive ability.

Our findings provide the first molecular evidence for substantial genetic influence on variation in children's educational achievement and its association with family SES. We further show that children's intelligence accounts for one third of this SNP link between family SES and children's educational achievement. In addition, we demonstrate that a GPS based on years of education in adulthood discovered in an independent large GWA meta-analysis^13^ significantly explains variance in children's educational achievement in our sample, even after controlling for intelligence.

## Materials and methods

### Sample and genotyping

The sample was drawn from the Twins Early Development Study (TEDS), a multivariate longitudinal study that recruited over 11 000 twin pairs born in England and Wales in 1994, 1995 and 1996.^28, 29^ TEDS has been shown to be representative of the UK population.^30^ Supplementary Table 2 shows that the genotyped subsample of TEDS is representative of UK census data from first contact through age 16 years.

The project received approval from the Institute of Psychiatry ethics committee (05/Q0706/228) and parental consent was obtained before data collection.

DNA data were available for 3747 children whose first language was English and had no major medical or psychiatric problems. From that sample, 3665 DNA samples were successfully hybridized to Affymetrix GeneChip 6.0 SNP genotyping arrays (Affymetrix, Santa Clara, CA, USA) using standard experimental protocols as part of the WTCCC2 project (for details see Trzaskowski *et al.*).^31^ In addition to nearly 700 000 genotyped SNPs, more than one million other SNPs were imputed from HapMap 2, 3 and WTCCC controls using IMPUTE v.2 software.^32^ A total of 3152 DNA samples (1446 males and 1706 females) survived quality control criteria for ancestry, heterozygosity, relatedness and hybridization intensity outliers. To control for ancestral stratification, we performed principal component analyses on a subset of 100 000 quality-controlled SNPs after removing SNPs in linkage disequilibrium (*r*^2^>0.2).^33^ Using the Tracy–Widom test,^34^ we identified 8 axes with *P*<0.05 that were used as covariates in GCTA and polygenic score analyses.

### Measures

#### Educational achievement

Educational achievement was operationalized as performance on the standardized UK-wide examination, the General Certificate of Secondary Education (GCSE), taken by almost all (>99%) pupils at the end of compulsory education at typically at the age of 16 years. English, mathematics and science are compulsory subjects. Five or more GCSEs with grades A*–C are required for further education, including GCSE English and GCSE mathematics. The joint performance on these three compulsory subjects determines admission to further education and employability.

The data for the present study were collected by questionnaires sent by mail and by telephone interview of parents and twins themselves. After completed forms were received from the families, the grades were coded from 11 (the highest grade: A*) to 4 (the lowest pass grade: G); no information about failed results was available. For 1729 individuals, self- and parent-reported GCSE results were verified using data obtained from the UK National Pupil Database,^35^ yielding correlations of 0.99 for mathematics, 0.98 for English and 0.96 for science.

The GCSE measure for the present analyses was the mean grade of the three compulsory core subjects, mathematics, English (mean grade of ‘English Language' and ‘English Literature'), and science (mean of any science subjects taken), requiring at least two measures to be nonmissing. Scores on the three compulsory core subjects were highly correlated (0.65–0.81).

#### Intelligence (IQ)

Individuals were assessed at the ages of 2, 3, 4, 7, 9, 10, 12, 14, and 16 years on general cognitive ability using a battery of parent-administered and phone- and web-based tests. At ages 2, 3, and 4, tests were parent-administered and validated against standard tests administered by a trained tester. At age 7, tests were administered over the phone; at age 9, parents administered the tests; and at the ages 10–16, tests were web based. At each testing age, individuals completed at least two ability tests that assessed verbal and nonverbal intelligence. Psychometric properties of the tests have been described in detail elsewhere,^36^ with the exception of the measurements used at age 16 years, where subjects completed a web-based adaptation of Raven's Standard and Advanced Progressive Matrices and the Mill-Hill Vocabulary Scale.^37, 38, 39^

For each composite measure at each of the nine ages, scores were regressed on sex and age, outliers above or below 3 s.d. from the mean were excluded and the standardized residuals were quantile normalized. Subsequently, a mean composite scale was created as the mean across the nine ages, performing mean-imputation for missing measurement occasions to avoid list-wise deletion.

#### Family SES

Converging evidence suggests that a composite of variables including parental education and occupation represents SES better than any single indicator.^18^ To index family SES, we combined parental education and occupation assessed when children were aged 2, 7 and 16 years. At age 2 years, SES was constructed as the mean of mother's and father's highest education level, mother's and father's occupation assessed by the Standard Occupational Classification 2000,^40, 41^ and maternal age at birth of eldest child. The SES composite when children were age 7 years was created similarly but without the variable of age of mother at birth of eldest child. At age 16 years, SES was composed as the mean of household income, maternal and paternal education level and maternal and paternal occupation. Mean composites were standardized and quantile normalized. The correlations between these three SES estimates ranged from 0.70 to 0.77. To increase reliability and maximize sample size, the final measure of family SES for this study was created as the mean composite score of parental SES reported when children were aged 2, 7, and 16 years, performing mean-imputation for missing data points.

### Statistical analyses

#### GCTA

The GCTA model decomposes the trait variance into an additive genetic component (_G_) captured by the available SNPs (and correlated markers in linkage disequilibrium with the genotyped SNPs) and a residual component containing all nonadditive genetic variance, interaction effects, environmental factors, error variance and additive genetic variance that is not tagged by the sampled SNPs. Hence, the GCTA model estimates lower-bound additive genetic variance for both phenotypes (*V*_G_^GCSE^, *V*_G_^SES^); and the correlation between the additive genetic components (*ρ*_G_). The *ρ*_G_ is not biased in the same way *V*_G_ is. This is because the estimate of genetic correlation is a function of the ratio between SNP-tagged covariance and SNP-tagged variance that are biased to the same extent (that is, the estimates are subject to the same imperfect linkage disequilibrium between causal variants and genotyped SNPs) and hence cancel each other out.^42^

Using genome-wide SNP data, we estimate genetic variation and covariation from a representative sample of 3000 unrelated children. Our estimates were obtained by restricted maximum likelihood using the published algorithm for GCTA.^11^ GCTA estimates the proportion of phenotypic variance of a trait tagged by sampled SNPs by fitting the polygenic effects of all SNPs simultaneously as random effects in a mixed linear model using a restricted maximum likelihood function. The so-called genetic relatedness matrix holds the mean pairwise genomic similarity (weighted by allele frequency) between all pairs of individuals in the sample across all SNPs. The variance tagged by all SNPs is estimated to be >0 when genetically more similar individuals are phenotypically more similar. The bivariate extension of the model relates the pairwise genetic similarity matrix to a phenotypic covariance matrix between traits (here family SES and educational achievement).^17^ To prevent confounding of the SNP estimate by shared environment effects and the effects of causal variants that are not tagged by the SNPs, cryptic relatedness was removed from the analyses. This default procedure eliminates one individual from a pair whose genetic similarity is 0.025 or greater; a coefficient that approximates at least fifth-degree relatives. The removal of close relatives ensures that estimates reflect the tagging of causal variants through population linkage disequilibrium. This criterion removed seven individuals from the analyses. Analyses were executed using GCTA^11^ and R software.^43^

The present sample size of ~3000 yields 80% power to detect a GCTA heritability estimate of 30% (α=0.05) and genetic correlation estimate of 0.6 (α=0.05; *V*_G_^1^=0.20; *V*_G_^2^: 0.30; r_Ph_=0.50).

#### Polygenic scores

We created polygenic scores from genome-wide data of over 3000 unrelated children using GWA results for total years of schooling from an independent discovery sample.^13^ The same quality control criteria as for the GCTA analyses were applied to the data. Polygenic risk scores were constructed using the *P*-values and β-weights from the recent large (*N*=126 559) GWA based on years of education.^6^ Quality-controlled SNPs were pruned for linkage disequilibrium based on *P*-value informed clumping in PLINK,^44^ using *R*^2^=0.25 cutoff within a 200-kb window. We removed the major histocompatibility complex region of the genome because of its complex linkage disequilibrium structure. 144 890 SNPs survived linkage disequilibrium pruning. For each individual, multiple polygenic scores were generated using the PLINK score option based on the top SNPs from the GWA analysis of educational attainment for varying significance thresholds (from 0.01 to 0.50). Numbers of SNPs per threshold are summarized in Supplementary Table 3. The scores were calculated as the sum across SNPs of the number of reference alleles for each SNP multiplied by the effect size (β-coefficient) derived from the GWA analysis of years of education.

Polygenic scores were tested for association with the same quantitative measures used in the GCTA analyses (family SES, educational achievement (GCSE), intelligence and educational achievement controlled for intelligence) in linear regressions. These analyses were corrected for the first eight ancestry-informative principal components by entering them as covariates into the regression models. Analyses were performed in PLINK and R.

## Results

Phenotypically, children's educational achievement correlated 0.50 (0.02 s.e.) with their family SES. Both variables also correlated with intelligence: 0.55 (0.02 s.e.) for educational achievement and 0.38 (0.02 s.e.) for family SES (Supplementary Table 1).

### Bivariate GCTA

**Figure 1:**
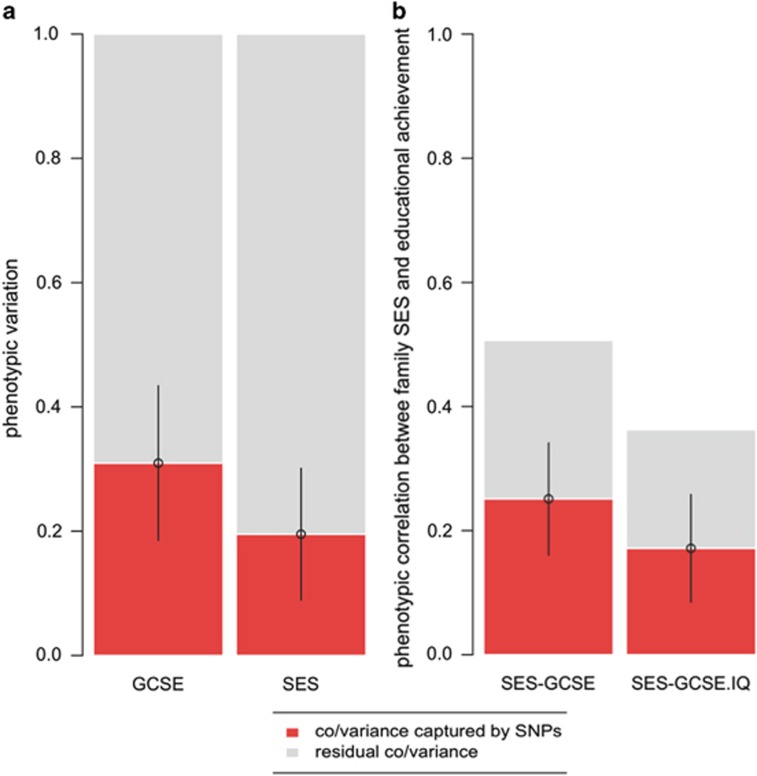
Bivariate genome-wide complex trait analysis (GCTA) of family socioeconomic status (SES) and children's educational achievement (General Certificate of Secondary Education (GCSE)). (**a**) Proportion of phenotypic trait variance tagged by the sampled SNPs in GCSE and family SES, respectively. (**b**) Covariance between family SES and GCSE captured by SNPs, without controlling for intelligence (left bar) and when controlling for intelligence (GCSE.IQ) (right bar). The length of the bar indicates the total phenotypic correlation between SES and GCSE. Solid black lines indicate standard errors.

Bivariate GCTA showed that the estimated proportion of variance tagged by the sampled SNPs was 0.31 (0.12 s.e.) in educational achievement, and 0.20 (0.11 s.e.) in family SES (Figure 1). The genetic correlation, indicating the extent to which the same SNPs are associated with family SES and children's educational achievement, was near unity (*r*_G_=1.02 (0.25 s.e.)).

Based on the genetic correlation between the two traits and the genetic contribution to variance of each trait respectively, GCTA estimates the genetic contribution to the phenotypic correlation between the two traits: *C*(*_G_*)=*r*_1,2_ (*_G_*) √ (*V*_1_ (*_G_*) × *V*_2_ (*_G_*)), applied to the data: 0.25=1.02 × √ (0.31 × 0.20). Hence, GCTA estimated the genetic contribution to the phenotypic correlation between family SES and children's educational achievement as 0.25 (0.09 s.e.), indicating that the proportion of the observed correlation tagged by the additive effects of available SNPs was 50% (that is, 0.25/0.50; Figure 1). This suggests approximately half of the phenotypic correlation between children's family SES and their educational achievement was mediated genetically.

#### Mediation analyses

To test whether intelligence mediates the observed association between family SES and children's educational achievement, we statistically controlled for intelligence by regressing GCSE on intelligence and entering the resulting standardized residuals into the bivariate GCTA model with family SES. When controlling for variance explained by children's intelligence, which yielded a univariate GCTA estimate of 0.38 (0.11 s.e.) (data not shown), the phenotypic correlation between family SES and children's educational achievement was reduced from 0.50 to 0.37 (0.02 s.e.). The GCTA estimate of the genetic covariation between family SES and children's educational achievement dropped from 0.25 (0.09 s.e.) to 0.17 (0.09 s.e.). Mirroring the mediation observed at the phenotypic level, this suggests that one-third of the SNPs tagging variation in family SES and children's educational achievement also captured individual differences in intelligence, implying two-thirds of the SNPs linking family SES and children's educational achievement were independent of intelligence.

### Polygenic score analyses

**Figure 2:**
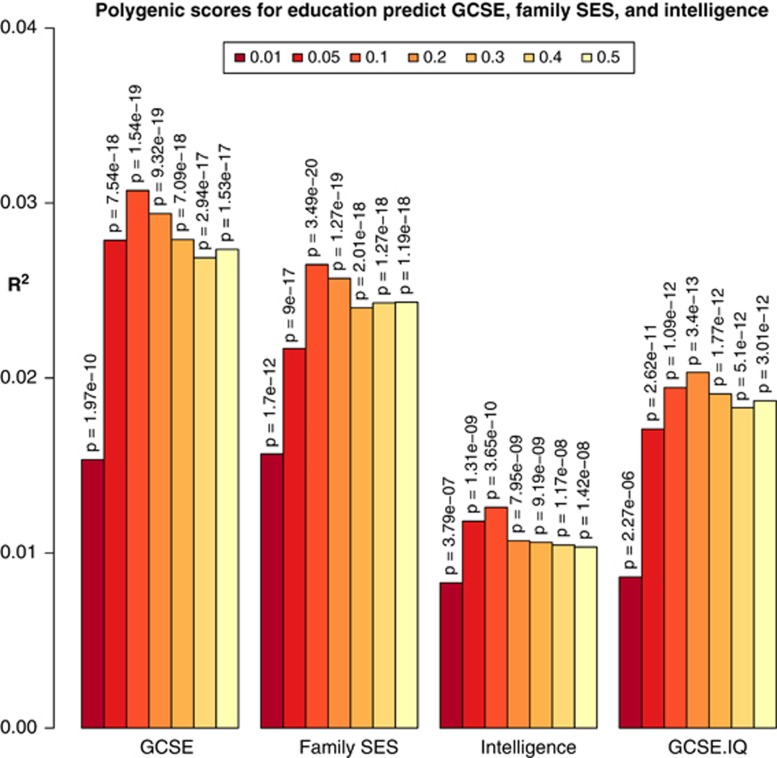
Genome-wide polygenic scores (GPS) for years of schooling in adults (Rietveld *et al.*^13^) predict variance (*R*^2^) in children's educational achievement (General Certificate of Secondary Education (GCSE)), family socioeconomic status (SES), intelligence and educational achievement after controlling for intelligence (GCSE.IQ). GPS were created using different significance thresholds for inclusion of variants for years of education, ranging from *P*=0.01 to 0.50, indicated by heat colors. The uncorrected *P*-values above each bar indicate the statistical significance of the observed association between the GPS and the respective trait.

Polygenic score analysis is designed to test whether SNPs that do not reach genome-wide significance in a discovery GWA are nonetheless significantly associated in aggregate with a trait in an independent sample. In the same sample of 3152 unrelated individuals, we created polygenic scores with varying numbers of SNPs (see Materials and methods) based on a large meta-analytic GWA study (*N*=126 599) of years of education.^13^ Figure 2 displays the results of multiple linear regression analyses showing that the polygenic scores accounted for ~3.0% variance in educational achievement (GCSE), ~2.5% in family SES and ~1.0% in intelligence. All *P*-values were ≤3.79^−07^. Notably, the effect size for GCSE remained substantial (~2.0%) and significant (*P*≤2.27^−06^) when statistically controlling for intelligence.

## Discussion

This study provides the first molecular evidence for substantial genetic influence on differences in children's educational achievement at the end of compulsory education in the United Kingdom and its association with family SES. Our GCTA results show that SNPs that are associated with both family SES and GCSE scores account for about half of the phenotypic correlation between SES and GCSE. Mediation analysis suggests that about one-third of this genetic effect also extends to children's intelligence, but two-thirds of the genetic association between family SES and GCSE scores is independent of intelligence. In GPS analysis, we show that SNPs associated with total years of education in adulthood discovered by an independent large GWA meta-analysis^13^ explain up to 3% of the variance in children's educational achievement in our sample, and up to 2% of the variance after controlling for intelligence.

The GCTA heritability estimate of 31% for children's performance on a UK national examination at the end of compulsory education corroborates the vast literature of traditional family-based methods, mostly the twin method, showing that variation in children's educational achievement is under substantial genetic influence,^4, 5, 7, 8, 9, 45, 46^ with heritability estimates converging at ~50%. This commonly observed discrepancy in phenotypic variance explained by pedigree-based methods (that is, twin and family) and population-based methods (that is, GCTA) occurs because GCTA only captures genetic variance contributed by additive effects of common SNPs that are in sufficient linkage disequilibrium with the causal DNA variants.^47^

Our GCTA heritability estimate of 20% for family SES tagged by children's genotypes is very similar to GCTA heritability estimates of years of education in adulthood and socioeconomic measures tagged by adults' genotypes themselves in previous studies.^13, 14, 15^ This is remarkable as children's genotypes are only a proxy for their parents' genotypes. In other words, GCTA effects on family SES estimated from children's DNA only reflect the extent to which children inherit parental characteristics associated with the family SES created by the parents. One such factor is intelligence, and we find that children's intelligence accounts for about one-third of the GCTA association between family SES and children's educational achievement. However, it is interesting that two-thirds of the GCTA association is *not* accounted for by children's intelligence. This finding of intelligence-independent shared genetic variance between family SES and children's educational achievement suggests that differences in educational achievement at the end of compulsory education and the level of education and occupation attained in adulthood are not merely the manifestation of differences in intelligence. This is in line with twin research that suggests that the heritability of educational achievement reflects many genetically influenced traits such as personality and self-efficacy, not just intelligence.^48^

The polygenic nature of behavioral traits poses a statistical challenge as enormous sample sizes are needed to identify genome-wide significant single DNA variants.^23^ Therefore, genome-wide methods, such as GCTA and GPS analysis, that aggregate genetic effects across a multitude of markers have the assumption of polygenicity at their core and provide powerful approaches for exploring genetic influences on traits and shared between traits.

A GPS based on markers associated with years of education in adulthood in an independent discovery sample was significantly associated with children's educational achievement in our sample. Replicating results from polygenic score analyses of a recent Dutch study,^49^ this shows that the shared polygenic link between children's educational achievement and adult measures of education even holds when limited to education-associated SNPs identified in an independent sample of adults. We further demonstrate that this polygenic link persists independently of children's cognitive ability, and that the educational attainment GPS of children's genotypes explains variance in their parents' socioeconomic status. The predictive power of GPS analysis in our independent sample illustrates that adequately powered GWA studies can identify replicable genetic associations with behavioral traits. Although the current GPS accounts for only a small amount of phenotypic variance, as prediction improves, GPS can identify profiles of genetic risk and protective factors for unrelated individuals, which will enable more powerful prediction models that combine genetic and nongenetic factors. Polygenic predictors might also facilitate research on the causal pathways underlying these genetic predictors.^21, 22, 50^

The results need to be interpreted in the context of three main important methodological limitations. First, a specific limitation of this study is its modest statistical power in the GCTA analyses (see Materials and methods). The GPS analyses were sufficiently powered to identify trait-associated variance at high statistical significance, but were limited by the power of the discovery GWAS to detect the small effect sizes of single variants across the genome.^21, 23^ A second, general limitation is the allelic spectrum covered by the current DNA microarrays, such as the Affymetrix 6.0 GeneChip used in our study, that is restricted to common variants. Research has begun exploring the relative contribution of common and rare variants to variation of psychiatric traits (see, for example, Gaugler *et al.*^51^ and Yang *et al.*^52^). Future studies with greater statistical power may explore the relative contribution of common and rare variants to trait variation of educational achievement and associated phenotypes. Third, both GCTA and GWAS, on which GPS analysis relies, are limited to detecting additive genetic variation that is captured by the sampled SNPs, which are typically common SNPs with minor allele frequencies >0.05. Hence, GCTA heritability provides a lower-bound narrow-sense heritability estimate and represents the upper limit for detection of SNP associations in GWA studies and thus for GPS analysis. Generally, these limitations imply a substantial underestimation of ‘true heritability' in the present analyses.

The present analyses demonstrate the ability of DNA-based methods to explore the genetic architecture of extended phenotypes such as family SES that cannot be detected by traditional variance/covariance estimation methods that rely on known kinship relatedness. Quantitative DNA-based methods, which rely on empirically established pairwise genomic similarity among traditionally unrelated individuals, can supplement and extend family-based methods and thereby facilitate the move from behavioral genetics to behavioral genomics.

Importantly, no directionality or causality can be inferred from the present results. Heritability indexes the proportion of trait variance attributable to genetic effects in a particular population at a particular time.^53^ Finding evidence for heritability of a trait or co-heritability of two traits does not imply resistance to environmental factors as genetic effects are dynamic and subject to developmental and environmental change.^54^ Research on how the heritability of educational achievement differs across development and across context suggests that genetic influences on these phenotypes are maximized by environmental opportunity.^54, 55, 56^ Differences in individuals' exposure to environments are not random. Genotype–environment correlation refers to the empirical observation that individuals experience different environments as a systematic function of their genotypes.^56, 57, 58, 59, 60, 61^ Genetic effects on phenotypes may be mediated through developmental or socio-contextual processes.

Our results also contribute to the extensive debate about meritocracy and social mobility^62^ that has largely ignored the fact that parents and their offspring are genetically related. Usually a lower correlation between parental and offspring SES is seen as an index of social mobility.^63^ However, considering genetics, we know that removing environmental sources of variation will not remove genetically driven resemblance between parents and offspring. To the contrary, as environmental differences diminish, individual differences that remain will to a larger proportion be due to genetic differences; that is, heritability would increase, which has also been demonstrated empirically.^55^ That way, heritability could be seen as an index of social mobility.

No necessary policy implications arise from finding heritability of educational achievement and its link with family SES. However, consideration of empirical evidence will lead to better-informed policy decisions. Specifically, analogous to the long-established model of evidence-based medicine, we believe that evidence-based education facilitated by a dialog between scientists and policy makers will be beneficial to education of all children and can also benefit schools, teachers, and society at large.^64^

In summary, our GCTA results show a substantial contribution of common SNPs to variation in children's educational achievement and its association with family SES. This is further substantiated by the GPS analyses, revealing significant sharing of genetic variants between children's educational achievement and total years of education in adulthood. Together, these findings provide converging evidence for substantial genetic influence on differences in children's educational achievement and genetic links with family SES. Our findings add weight to the view that genetic variation plays an important, but not exclusive, role in educational inequalities and social mobility, which is at variance with views, that still prevail in some quarters, that these are solely the product of social forces and environmental inequalities.
