# Supplementary material for: Genetic link between family socioeconomic status and children's educational achievement estimated from genome-wide SNPs
Source: Mol Psychiatry. 2015 Mar 10;21(3):437–43. doi: 10.1038/mp.2015.2 (PMC4486001; doi:10.1038/mp.2015.2)
Supplement: Supplementary Table 1 [file mp20152x1.pdf]

	GCSE	Family SES	Intelligence
**GCSE**	**1**		
N	2603		
**Family SES**	**0.50 (0.02)**	**1**	
N	2583	3115	
**Intelligence**	**0.55 (0.02)**	**0.38 (0.02)**	**1**
N	2560	3055	3089
