# Supplementary material for: Genetic link between family socioeconomic status and children's educational achievement estimated from genome-wide SNPs
Source: Mol Psychiatry. 2015 Mar 10;21(3):437–43. doi: 10.1038/mp.2015.2 (PMC4486001; doi:10.1038/mp.2015.2)
Supplement: Supplementary Table 2 [file mp20152x2.pdf]

	Returned data (N families)	% parents A-levels or higher	% Mother employed	% Father employed	% Female
UK census[a]	--	32%	49%	89%	50%
TEDS 1st contact	3,152	29%	47%	89%	54%
TEDS age 16	2,886	31%	47%	89%	55%

a The UK 2001 census data (ONS, 2001) for families with children were used because they represent more appropriate comparisons than UK 2011 census data for TEDS twins who were born 1994 -1996.
