# Supplementary material for: Genetic link between family socioeconomic status and children's educational achievement estimated from genome-wide SNPs
Source: Mol Psychiatry. 2015 Mar 10;21(3):437–43. doi: 10.1038/mp.2015.2 (PMC4486001; doi:10.1038/mp.2015.2)
Supplement: Supplementary Table 3 [file mp20152x3.pdf]

p_T	Number of SNPs
0.01	5,733
0.05	19,333
0.10	32,398
0.20	54,250
0.30	72,745
0.40	88,960
0.50	103,009
