# Supplementary material for: Genetic link between family socioeconomic status and children's educational achievement estimated from genome-wide SNPs
Source: Mol Psychiatry. 2015 Mar 10;21(3):437–43. doi: 10.1038/mp.2015.2 (PMC4486001; doi:10.1038/mp.2015.2)
Supplement: Supplementary Table Legends [file mp20152x4.doc]

**Supplementary Table 1.** Phenotypic correlations (Pearson’s *r*), including standard errors and sample sizes (individuals entered into GCTA analyses, i.e. genotyped individuals who survived quality control).

**Supplementary Table 2.** The genotyped subsample of the Twin Early Development Study (TEDS) is representative of UK census data from first contact through age 16.

**Supplementary Table 3.** Number of SNPs per total years of education *p*-value threshold (P_T_) used for genome-wide polygenic score creation.
